# When is your experience valuable? Occupation-industry transitions and self-employment success

**DOI:** 10.1007/s00191-017-0528-2

**Published:** 2017-08-19

**Authors:** Sierdjan Koster, Martin Andersson

**Affiliations:** 1 Faculty of Spatial Sciences, University of Groningen, Groningen, Netherlands; 2 Department of Industrial Economics, Blekinge Institute of Technology (BTH), Karlskrona, Sweden; 3 Research Institute of Industrial Economics (IFN), Stockholm, Sweden; 4 Swedish Entrepreneurship Forum and CIRCLE, Lund University, Lund, Sweden

**Keywords:** Entrepreneurship, Self-employment, Occupational choice, Human capital, Skills, Spin-offs, Experience, L26, J24, J62, J23

## Abstract

The literature on employee spinoffs has, for a long time, stressed the importance of industry-specific skills and experiences in explaining the success of new firms. We argue that employees also develop skills that are associated with their occupation within an industry, and that success as an entrepreneur, therefore, is also contingent on the relation between the entrepreneurs’ previous occupation and the industry in which they operate as self-employed. Using matched employer-employee data, we develop a measure, occupational spin-offs, that accounts for this relation. An occupational spin-off is defined as a start-up in the most common industry, given the previous occupation of the founder. We then show that entrepreneurs starting occupational spinoffs enjoy above average income from self-employment and have longer spells as business owners.

## Introduction

Self-employment success importantly hinges on the human capital available to the business owner. Part of the human capital is built up in the course of a labor market career. People develop skills, build up relevant professional networks and acquire problem solving capabilities. They can capitalize on the combined stock of experiences available to them by starting their own firms. The specificity and quality of these experiences are important determinants of self-employment success (Phillips 2002). In addition, the range of the skills is important. Successful business owners tend to rely on a wide variety of skills, which allows them to oversee all aspects of the business. This view is established in the so-called ‘jack-of-all-trades’ theory as developed by Lazear (2004) and empirically tested by Bublitz and Noseleit (2014), among others. Unger et al. (2011) argue that human capital is most valuable in relevant contexts, i.e. when it is task-specific. Two persons with identical skill sets may experience very different outcomes, depending on the context in which the human capital is deployed. The correspondence between the context of on-the-job learning and the context of deployment is thus an important aspect in explaining and understanding heterogeneity between entrepreneurs in their success in entrepreneurship and self-employment.

In explaining self-employment success, the correspondence between the learning context as an employee and the context of deployment in entrepreneurship has mostly focused on the industry dimension. This follows a large literature on spinoffs and organizational heredity, which shows that new firms that start in the same industry as the parent organization tend to perform better than other types of start-ups in terms of both growth and survival. There is empirical evidence in this regard from studies with firms as the unit of analysis (Klepper 2001), as well as from studies with individuals as the unit of analysis (Hamilton 2000). Hence, self-employed with same-industry experience are likely to outperform those who lack such specific experience. Learning, however, is multi-faceted and part of on-the-job learning may be occupation-specific rather than industry-specific (Kambourov and Manovskii 2009). As a result, focusing on same-industry experience alone clouds considerable underlying heterogeneity in on-the-job learning.

This study expands the existing literature by not only assessing the role of same-industry experience in self-employment success, but also regarding the effect of the correspondence between the occupational background and the industry in which the self-employed is active. The intuition of the idea is illustrated by the fact that many firms have a strong division of labor and consequent specialization of tasks. Large manufacturing firms such as Ericsson, ABB, Philips, and Unilever typically have a broad set of employees with specialized skills in occupations that, in themselves, would not classify as manufacturing as such. Examples include software development, accounting and human resources. The experiences built up in these types of occupations likely better prepare for self-employment in, respectively, the IT-sector, financial services and business services than for self-employment in manufacturing. This shows that the relation between tasks performed and industry may be rather detached. Addressing this issue, Kambourov and Manovskii (2009) remark that industry classifications were never meant to reflect differences in learning. To account better for on-the-job learning, the occupation is relevant (Gathmann and Schönberg 2010). We apply this idea to self-employment.

The occupational background of individuals who switch to self-employment is important in two distinct ways. First, certain occupational groups have a higher propensity of moving into entrepreneurship (Lazear 2004; Bublitz and Noseleit 2014). Managers, for example, possess general and easily transferable skills that prepare them well for entrepreneurship.^1^ Transition rates into entrepreneurship are thus expected to vary across occupational groups. Second, over and beyond the selection effect into entrepreneurship, we postulate that a relevant occupation-industry combination may be positively related with entrepreneurial success. To account for this, we identify the most common start-up industry given a certain occupational background. In doing so, we assume that those occupation-industry combinations reveal the closest match between a certain occupational background and the skills relevant to an industry. The firms started by entrepreneurs that make this most common transition are labeled ‘occupational spin-offs’, similar to the generic term spin-offs that denotes firms started by entrepreneurs with same-industry experience (See, Garvin 1983; Klepper 2001). In analogy to Neffke and Henning (2013) - who use between-industry worker flows that are over and beyond expected worker flows given the respective sizes, growth rates and wage levels of the industries -, we argue that transitions from occupations as employees to industries as self-employed reflect ‘revealed-relatedness’ between the two.

We analyze a detailed matched employer-employee database for Sweden that allows us to identify employees who choose to leave their job for entrepreneurship (Andersson and Klepper 2013; Hyytinen and Maliranta 2008). We restrict this transition to entrepreneurs who go from being fully employed to being fully self-employed. In addition, only new firms with sole ownership are included. By imposing such restrictions, potential unobserved and confounding skill sets or resources that may be available to the new firm through a current employer (in the case of hybrid start-ups) or team members (group start-ups) are eliminated from the analysis.

The main findings are that occupational spin-off entrepreneurs have higher success, as evidenced by higher business income and longevity as entrepreneurs. These results are robust to the inclusion of a full set of theoretically motivated control variables that account for other modes in which relevant experience is gained (including employer size, job tenure, age and industry) and other controls for entrepreneurial success (including migration, regional context). In addition to the main conclusion of the study regarding the pertinent positive effect of a relevant occupational background, the results confirm recent findings in the literature regarding the effects of the variables mentioned in the above. The intuition of the results is that the occupational background of self-employed is an important element in representing the multi-faceted nature of learning-on-the-job processes.

Our analyses contribute to the existing literature on the relationship between firm-specific (Becker 1964) and industry-specific learning (Klepper 2001) and built-up experiences and entrepreneurial success. As such, the argument speaks to important debates around the type of contexts that stimulate the recognition of entrepreneurial opportunities and the decision to act successfully on them (Shane and Venkataraman 2000; Shane 2003). These underlying mechanisms are crucial in understanding the heterogeneity in entrepreneurship, both in its manifestations and its success. The findings also suggest that descriptions of regional specialization and diversification can be meaningfully extended beyond industry-structure to also include information on the occupational structure of the regional economy.

## Explaining self-employment success

In the explanation of self-employment success, the availability of skills and experiences available to business owners takes center stage. This notion is derived from the labor market literature that links the development of human capital to labor market success. In this respect, Gary Becker (1964) provides an important account of human capital. He describes the ways in which individuals gather human capital and how productivity and earnings are influenced as a result. In this approach, employment or self-employment is not a static individual feature, but a temporary state that must be seen in the context of the whole working career. Spells of employment, or self-employment, give individuals the possibility to develop their skills to reach a higher level later in their career (Burke et al. 2008). This means that the current labor market status of an individual can also be a result of previous activities. People develop skills and transfer them to new settings, either as employees or as entrepreneurs. The skills are most valuable when they are transferred to a suitable environment; a carpenter cannot use skills specific to carpentry in a bakery. The correspondence between the learning environment and the context of deployment is thus key. To conceptualize the transferability of skills, Becker makes the distinction between general training and specific training. General training entails the development of skills, either through training per se or through experience, which are also useful outside the firm that provides the training (Becker 1964, p.11). General skills can be deployed in a range of settings. Management skills are a clear example, but also marketing skills may fall in this category. In contrast, specific skills have a greater positive effect for the providing firm than for other firms. In other words, general training results in skills that are easily deployed in other firms, whereas specific skills lose their merit outside the context of the source firm. It is not a clear dichotomy, however. The two extremes form the poles of a spectrum, and most training will be between the two extremes. Becker (1964, p.18) suggests that purely specific training is unlikely to occur and there is always a possibility to transfer the specific skills. In particular, firms that are similar to the source firm can benefit.

In Becker’s original framework, the firm is the context for specific learning. The framework can, for example, explain why employees with a longer tenure earn more on average. They have developed firm-specific skills that make them more valuable to the firm, all else equal. The dimension of the firm, in terms of specific learning, seems less applicable in the case of self-employment success as the employees have, by definition, left the firm. Firm-specific skills, in its extreme form, are thus rendered useless. However, there are additional dimensions along which specific training can be conceptualized: industry-specific skills and occupation-specific skills.

In explanations of self-employment success, as well as success in new firms in general, industry skills have long been recognized. Wennberg et al. (2011), for example, compare the performance of corporate and university spinoffs and find that performance of the former is greater. One explanation of this is that university spinoffs may have lower industry experience. This interpretation is further supported by the finding that prior industry experiences are more important for university spinoffs that are founded by individuals lacking the corporate context. The general argument is that people active in a certain industry build up skills that lose part of their merit when transferred outside of the boundaries of the industry. As a result, employees have a strong incentive to stay in the same industry when moving into self-employment and they are likely to outperform entrepreneurs without industry-specific experience. This is the main argument in the literature on spin-offs, which tends to confirm that spin-off performance is above average, both at the firm level (for example, Klepper 2001) and at the level of the business owner (Sørensen and Phillips 2011; Iversen et al. 2010). Shane (2003) stresses that industry experience is important in the recognition and evaluation of business ideas. As such, those with industry-specific skills and experiences are likely to develop more viable business ideas reflected in the increased performance of the new firm. Likewise, Neal (1995) and Parent (2000) demonstrate the effect of industry experience in the context of wage employment. Displaced workers finding a job in the same industry in which they previously worked, experienced higher wages - or a smaller wage drop – than those switching industries.

In addition to learning in the context of the firm and the industry, learning can be occupation-specific (Kambourov and Manovskii 2009). Occupations can be conceptualized as combinations of tasks and Gathmann and Schönberg (2010) show that mobility between occupations that are similar in terms of task combinations is associated with higher wage gains than mobility between occupations that are further apart. This suggests that occupations are appropriate units for studying task profiles, even though the underlying task profiles themselves may be masked. Showing the relevance of occupation-specific human capital, Kambourov and Manovskii (2009) demonstrate that occupational tenure is an important driver of wage growth. They find that five years of occupational tenure is associated with a wage increase of 12% to 20%. This effect supersedes the effect of industry and firm tenure, indicating that occupational background is a pertinent dimension of specific learning in the context of wage employment.

The aspect of occupation-specific skills has gained limited traction in explanations of self-employment success. Yet, it is likely a relevant dimension along which employees build up skills that can be used in self-employment. It represents an additional dimension of learning that is not necessarily captured by industry experience. Industry experience as a measure of skills and experiences pertinent to an entrepreneur can thus conceal a large amount of heterogeneity in the tasks and the associated learning processes. Consider an IT-specialist working in a manufacturing firm. Part of the relevant skills and experiences may be industry-specific and thus picked up in a conventional spin-off definition. However, it is very likely that part of the specific learning is mainly relevant in the IT-sector. Relevant networks, for example, may well be located in the IT-sector rather than in the manufacturing industry. The skills developed by the employees may also be mainly relevant in the IT-sector.

Against this backdrop, we suggest that (i) different occupations are associated with different skills and experiences, (ii) those skills and experiences are not equally deployable in all industries, (iii) therefore, for a given occupational background, some industries are more relevant than others. As a result, (iv) the success of entrepreneurs in an industry may be higher if the entrepreneurs have a relevant occupational background. Such occupation-industry links between wage employment and self-employment reflect the idea that the value of human capital depends on the context in which it is used (Unger et al. 2011).

This type of occupation-specific learning is over and above the more general notion that certain occupations are effective learning environments for self-employment as such. Specifically, the jack-of-all trades theory suggests that occupations with a balanced skill set may be appropriate backgrounds for self-employment (Lazear 2004; Bublitz and Noseleit 2014). Also, some occupations may involve tasks that are relatively easily adopted in different contexts. This can explain why transitions into self-employment are more common for certain occupations. As a case in point, Lazear (2004) shows that most self-employed have a background in an executive, administrative or managerial position (33%), well above their overall share in the economy.

Occupation-specific skills can thus be pertinent to both the practice of being a business owner, which can be thought of as another occupation, and to the content basis of the firm. The latter is represented by the industry in which the business owner is active. Common occupation-industry combinations reflect the relevancy of occupation-specific learning in a certain industry.

### Occupational spin-offs

The theoretical arguments concerning specific learning suggest that starting a business in an industry related to the previous occupation of the entrepreneur affects performance. Testing this hypothesis requires a correspondence between occupations and industries, but such a correspondence does not exist or is not readily available.

To define occupational spinoffs, we build conceptually on the principle of *revealed relatedness* (Neffke and Henning 2013). Neffke and Henning (2013) argue that relatedness between industries is revealed by labor flows, because, when switching jobs, individuals are expected to remain in industries that value the skills they developed in their previous work. Gathmann and Schönberg (2010) demonstrate that this principle also holds for occupations and they empirically show that mobility between occupations is more likely between occupations with a similar task portfolio than random mobility between occupations would suggest. Worker flows can thus be meaningfully used to indicate relatedness.

In our empirical context, entrepreneurs are expected to start a firm in the industry in which they have the greatest opportunities to exploit the experiences and skills they have obtained previously. For entrepreneurs with a given occupational background, we argue that their typical choice of industry in which to start their new firms should inform about the relatedness between the occupation as employee and industries as self-employed. The most common industry of choice for entrepreneurs with the same occupational background reflects the industry in which the experiences obtained in the prior occupation are most frequently valuable. Based on these arguments, we will define occupational spin-offs as follows:

“An occupational spin-off is a new firm started in an industry that corresponds to the most common industry of choice for entrepreneurs with the same occupational background as the founder.”

## Data and empirical strategy

### Data and definition of self-employed

The data for the study are a matched employer-employee dataset for Sweden for the period 2004–2009. These data comprise all establishments, firms and employed individuals in the country. Each individual’s employer (establishment/firm) is determined annually by his place of work in the month of November. The information for each employee is extensive and includes age, gender, employment status, income, place of residence, place of work, education and occupation. We use these data to identify persons who switch from employment to self-employment between 2004 and 2005, and these entrepreneurs are then followed in the period 2005–2009.

We pose several restrictions on the transition from employment to self-employment. The restrictions are aimed at reducing the influence of potential skill-sets available to a new firm other than that of the owner. First, we restrict the data to ‘pure’ employees in 2004, thus excluding employees with a business on the side. Entrepreneurs with recent experiences - in the year prior - as a business owner are thus eliminated. We focus on employees in the private sector, excluding agriculture, fishing and extraction sectors. Start-up dynamics in these sectors are highly specific given the inherent link to the physical resources used and the fact they are heavily regulated in Sweden. Also, specifically for farms, many turn to self-employment because they inherit the farm from their parents. The entrepreneurial start-up process is these sectors is thus different than the process we aim to address in the study. As entrepreneurs’ occupational backgrounds play an important part in the analysis, we restrict the data to those employees for which occupation information is available in 2004. Self-employed coming from a spell of unemployment are thus excluded. The sampling strategy suggests an overrepresentation of opportunity-based entrepreneurs who voluntarily choose entrepreneurship to capitalize on their experiences and skills. Pushed entrepreneurship as a result of closing firms is not ruled out, though, and it is accounted for in the empirical analysis. The restrictions leave us with 1,472,380 employees in 2004 working in firms with private owners in sectors 15–74 at the 2-digit NACE rev. 2 industry classification scheme.^2^ The original dataset for employees in 2004 includes about four million employees.

We then use information on the individuals’ employment status in 2005 and isolate individuals for whom the own firm is the only source of income in 2005. In this way, we do not count employees who start a business on the side of regular employment as switching from employment to self-employment. This limits issues regarding using the job in wage employment as an alternative source of skills. A further requirement is that entrepreneurs must be single owners of their businesses. The reason for this is that we aim to assess how the occupational background of the entrepreneurs influences performance and the empirical design is not appropriate for new firms with several owners who may have different occupational backgrounds. Finally, instances in which the step to self-employment is made by taking over an existing firm are excluded from the dataset (see also Andersson and Arvidsson 2011).

With these requirements, we end up with 3615 self-employed in 2005 who were regular employees in 2004; 2894 (80%) of these have no employees in the initial year, and another 10% have one employee. The bulk of our self-employed are thus small in terms of employees. The vast majority (70%) are single owners of new incorporated firms, whereas the rest are sole proprietorships. In line with the general trend that the service industry accounts for an increasing share of the economy (see Schettkat and Yocarini 2006 for an overview), most self-employed in our sample enter the broadly defined service industry (90%).

### Identifying occupational spin-offs

For establishing the relation between occupations and industries, we listed the number of start-ups across 2-digit NACE industries by the prior occupations that their owners held. The occupation classes correspond to the 1-digit ISCO-88 classification system. Even though this is a coarse approach, there are reasons to proceed in this way. First, the conceptual idea refers to broad characteristics of occupations. The more fine-grained the occupational categories become, the smaller the differences between the general task-composition of occupations. Second, the structure of the ISCO classification suggests taking this approach. The more disaggregated levels add industry information to the occupations (manager in a certain industry, for example). This would pollute the data on occupation specific skills and make it impossible to distinguish it from industry-specific learning. Third, the number of occupation-industry transitions decreases rapidly when using more fine-grained occupational categories, making the analysis vulnerable to idiosyncrasies.

We then identified the most common choice of industry, given a certain occupation by identifying the 2-digit industry with the highest number of new firms for each 1-digit occupation.^3^ We then create an ‘occupation spinoff’ dummy variable which is 1 if the industry in which the entrepreneur becomes self-employed is the most common for his previous occupation.^4^ The occupations all pertain to the year 2004 and the industries pertain to the year of start-up, 2005. Potentially relevant occupations before 2004 are thus excluded and the approach provides a conservative estimate of the number of occupational spin-offs. The result of the matching exercise is presented in Table 1.

**Table 1:** Most common industries of self-employed in 2005 by prior occupation (2004)

Occupation in 2004 (1-digit ISCO-88)	Most common 2-digit NACE code in 2005	Description of NACE code	Fraction in most common industry (%)	N
Legislators, senior officials and managers	74	Other professional, scientific and technical activities	41.9	611
Professionals	74	”	63.3	833
Technicians and associate professionals	74	”	34.7	718
Clerks	74	”	24.3	136
Service workers and shop sales workers	52	Warehousing and transport support	26.4	280
Skilled agricultural and fishery worker	1	Crop and animal production	54.6	11
Craft and related trades workers	45	Wholesale, retail of motor vehicles	54.7	576
Plant and machine operators and assembly	60	Programming and broadcasting activities	45.2	352
Elementary occupations	55	Accomodation	20.4	98
				3615

The table reports the most common 2-digit industries of entrepreneurs by occupational background according to the 1-digit ISCO-88 classification system. The NACE rev 2 system is used

The table shows that for all occupations except skilled agricultural and fishery workers, the most common 2-digit industry is in the broadly defined service industry, reflecting the fact that this is the most common industry in which to start a business. There are, however, differences across occupations in terms of which service industry is the most common. For entrepreneurs with more advanced prior occupations, such as managers, professionals and technicians, the most common industry in which they found their firms is industry 74, knowledge intensive business services. Almost 42% of all entrepreneurs that previously worked as legislators, officials and managers start in this industry. The corresponding numbers for professionals and the groups of technicians and associate professionals are 63% and 35%, respectively. We interpret this as reflecting that the tasks and functions undertaken by skilled employees in advanced occupations are relevant to business services.

Even though defined differently, there is potential overlap between occupational spin-offs and industry spin-offs. Are they empirically distinct? Table 2 addresses this issue and provides a simple cross tabulation of the two dimensions of experience, i.e. industry-industry and occupation-industry. The columns report those entrepreneurs who start a firm in the same two-digit industry in which they previously worked and those who change industries. This is a standard operationalization of industry experience in the literature on spin-offs (Garvin 1983; Klepper 2001). The other dimension is the occupation-industry dimension, as explained in the above and as presented in Table 1.

**Table 2:** Cross-tabulation of two types of experience; industry-industry and occupation-industry

	Same Industry	Different industry	*Total*
Occupational spin-off	1185	454	1639
Other	1029	947	1976
*Total*	2214	1401	3615

The table reports a cross-tabulation between occupational and industry spin-offs. Occupation spin-offs are defined as new firms starting in industries that corresponds to the entrepreneur’s previous occupation (Table 1), whereas industry spin-offs are new firms starting in the same 2-digit NACE industry in which the entrepreneur previously worked

Despite the broad occupation and industry classifications upon which occupational spin-offs are defined, fewer than half of the entrepreneurs are involved in an occupational spin-off. In total, out of all newly self-employed, 45% are identified as an occupational spin-off, whereas 61% can be labeled as an industry spin-off. The two dimensions appear to capture distinct processes in that they overlap only to a limited extent, as evidenced by the off-diagonal values. A fair share of entrepreneurs classified as occupational spin-offs start a firm outside their 2-digit industry (27%, 454/1639*100). Second, almost half (46%) of all industry spin-offs are started by people without relevant occupational experience by our definition. This indicates the underlying job heterogeneity within industry classifications.

### Empirical strategy

We assess the effect of the match between an entrepreneur’s occupational background and the industry in which he operates as an entrepreneur on self-employment success in two ways. First, we study the influence of the occupational spinoff dummy on business income generated from self-employment. As hybrid entrepreneurs are excluded from the data, this is a clean measure of the success as an entrepreneur. Still, the levels of income must be interpreted with caution as entrepreneurs have tax incentives to suppress the income generated. In the Swedish progressive tax system, such incentives are greatest in the upper ends of the income distribution, suppressing the difference between lower and higher income levels. Second, we analyze survival as a business owner. The dependent variable does not pertain to the success of the firm but rather to the success as an entrepreneur. It is important to address both the income from self-employment and the survival as an entrepreneur as they measure different aspects of success. Hamilton (2000) shows that people remain in self-employment even if the firm performs below expectations and the pecuniary returns are relatively low.

#### Business income

To analyze the influence of occupational spin-offs on business income, we formulate a two-stage selection model on the income generated from self-employment (Heckman 1979). This model is motivated on the grounds that those switching from employment to self-employment comprise a select group of people. Regressing business income on characteristics and experiences only based on individuals who are self-employed means that one does not observe the whole population. This creates a sample selection problem, as it is likely that those who choose to become entrepreneurs will tend to have higher business incomes than those who did not choose to become entrepreneurs would have had, had they chosen to become entrepreneurs. For example, it is likely that only those with special entrepreneurial skills or industriousness, and thus high expected business income, may choose to leave employment for self-employment. Heckman’s sample selection model deals with this issue by treating the selection problem as an omitted variable problem. Formally, the selection model can be formulated as follows (Puhani 2000):

1a $$ {y}_{i1}^{\ast }={\mathbf{x}}_{\mathbf{i1}}^{\prime }{\beta}_1+{\varepsilon}_{i1} $$

1b $$ {y}_{i2}^{\ast }={\mathbf{x}}_{\mathbf{i}2}^{\prime }{\beta}_2+{\varepsilon}_{i2} $$

$$ {\displaystyle \begin{array}{c}\hfill {y}_{i1}={y}_{i1}^{\ast}\kern1.75em \mathrm{if}\ {y}_{i2}^{\ast }>0\hfill \\ {}\hfill {y}_{i1}={y}_{i1}^{\ast}\kern2em \mathrm{if}\ {y}_{i2}^{\ast }>0\hfill \end{array}} $$

where ${y}_{i1}^{\ast }$ is the outcome of interest for individual *i*. The outcome is not observed for all individuals. ${y}_{i2}^{\ast }$is the selection variable, because we only observe the actual outcome for individuals *i*, *y*_*i*1_, for which ${y}_{i2}^{\ast }$> 0. Equation (1b) is thus a selection equation that singles out individuals for which we observe the pertinent outcome. The Heckman model is estimated in two stages. First, the selection equation is estimated, typically with a Probit model. Second, the outcome equation is estimated based only on those individuals for whom the outcome is observed, *y*_*i*1_ (Equation 1a). This second stage corrects for sample selection by including the Inverted Mills Ratio (IMR) computed from the selection equation in stage 1.^5^

In our empirical context, the outcome is income generated from self-employment, which is only observed for those individuals who move from employment to self-employment. Our selection equation is thus a Probit model in which we estimate the likelihood of switching from employment into self-employment, and this selection equation is based on the full sample of employees. Below we describe in detail which variables that enter into the respective equations and motivate their inclusion.

Importantly, the model should account for a variety of experiences when explaining self-employment success to identify the effect of occupational spin-offs over and above other aspects of learning. Throughout our empirical analyses, we consistently control for age, employer tenure, wage in the prior occupation and a traditional industry spin-off indicator. All these variables reflect different types of experiences. Age is perhaps the most generic indicator of general learning. Employer tenure can be assumed to capture firm- or employer-specific learning (Becker 1964). Wage in the prior occupation can be interpreted as a ‘catch-all’ variable related to learning specific to occupation, industry and employer. Illustrating this, Kambourov and Manovskii (2009) analyze the influence of occupational, industry and employer tenure, respectively, on an employee’s wage. Their finding is that all three aspects of tenure matter in explaining wages, but that occupation tenure appears to be more important than the other two. This suggests that prior wage is indeed a ‘catch-all’ variable, which may reflect all three types of learning. As is evident below, we control for prior wage in all our specifications. Finally, on top of these three measures, we include a traditional industry spinoff indicator in the form of a dummy which is 1 if the individual chooses to start his business in the same industry in which he previously worked. By consistently controlling for all four aspects of learning, we alleviate the risk that an estimated effect our variable of main interest, i.e. the occupational spinoff dummy, may capture some other type of learning that correlate with the occupational spinoff indicator.

##### Heckman selection model – The selection equation

We control for variables at three levels: (i) the level of the individual, (ii) the employer, and (iii) the local environment in which the individuals operate. We motivate and discuss the individual variables in each category below. Table 3 lists all variables in the analysis and their descriptive statistics.

**Table 3:** Variables and descriptive statistics

	Variable	Mean	Std. deviation	Min	Max
Individual characteristics	Self-employed (in 2005)	0.002	0.049	0	1
	Employees of self-employed	0.0004	0.022	0	1
	Age	39.444	10.837	20	60
	Age squared	1673.61	881.21	400	3600
	Male	0.668	0.470	0	1
	Highly educated	0.182	0.385	0	1
Employer/Job characteristics	Tenure	5.493	5.400	1	20
	Management	0.063	0.244	0	1
	Specialist	0.113	0.317	0	1
	Qualified	0.191	0.393	0	1
	Office	0.113	0.317	0	1
	Service	0.112	0.315	0	1
	Wage income (log)	7.792	0.608	0	12.346
	Size of employer (log)	4.100	1.952	0	12.455
	Establishment exit	0.025	0.155	0	1
	MNE	0.5707	0.494	0	1
	Hightech	0.047	0.212	0	1
	Medium hightech	0.122	0.327	0	1
	Medium lowtech	0.083	0.276	0	1
	Lowtech	0.105	0.306	0	1
	Knowledge Based Services	0.216	0.411	0	1
Local environment	Stayer	0.956	0.205	0	1
	Countryside	0.309	0.462	0	1
	City	0.281	0.449	0	1
	Metropolitan	0.389	0.487	0	1

At the individual level, we first control for age. As argued above, age is as a general indicator of experience and is expected to be positively related to the decision to transcend from employment to self-employment.^6^ However, with age increasing beyond a certain threshold, the time to earn back investments in the new firm may become too short. The opportunity cost of starting is then increasing. We therefore include the square of age, which we expect to have a negative impact on the probability for self-employment. Gender is controlled for as men are more likely to switch to self-employment (Cowling and Taylor 2001; Blanchflower and Oswald 1998). Furthermore, we include information on the education background of individuals in the form of a dummy variable indicating whether the employee finished a higher-level education program corresponding to a university degree of at least three years of study. The likelihood of switching to entrepreneurship has been found to increase with education (Rees and Shah 1986).

Since the general propensity to change from employment to entrepreneurship may differ across occupations, we include dummies for occupations involving skills that can be more generally adopted (*Management*) and those that indicate higher functional levels (*Specialist* and *qualified worker*). These are occupations that are expected to have a higher propensity of moving into self-employment compared to lower level occupations. Two additional individual-level controls are tenure (in years) at the workplace and wage income in 2004. As argued above, both variables could be said to reflect experience. However, they could also signify higher opportunity costs of self-employment. For example, labor economists frequently argue that tenure could be a proxy for the match quality between the employer and employee (Farber 1994), and employees may, in this case, be unlikely to leave their job for entrepreneurship.

At the level of the employer, we first account for the size of the previous firm. There is a long-standing discussion in the literature regarding the influence of employer size on self-employment (Hyytinen and Maliranta 2008; Elfenbein et al. 2010; Hvide 2009; Lazear 2004; Sørensen and Phillips 2011). A general finding is that employees in small firms are more likely to switch from employment to entrepreneurship. A main argument is that employees in small firms are more exposed to the whole business process, which makes them better equipped to start a firm. We also include a dummy variable indicating multinational enterprises (MNE). Andersson and Klepper (2013) find that employees in MNEs are less likely to leave the firm, which possibly reflects the idea that MNEs offer many opportunities for career progression within the boundaries of the MNE. Further employer-level controls include indicators for the industry in which the employer is active. The model includes dummy variables for high-tech, medium high-tech, medium low-tech and low-tech manufacturing industries, respectively, as well as a dummy for knowledge-based services. The general hypothesis in the literature is that more high-tech and knowledge-intensive sector should spur entrepreneurship, since the employees operate in an industry-context characterized by many entrepreneurial opportunities (Acs et al. 2009). A final employer-level control is a dummy for whether the establishment at which an individual works has closed down. The reason is simply that establishment closure could push employees into entrepreneurship.

Regarding the regional context of the entrepreneur, we control for whether the employees operate in metropolitan, city or countryside regions. A large literature on agglomeration economies argues that large and dense metropolitan regions stimulate entrepreneurship. One specific argument in this regard has to do with that such large regions offer thick local labor markets, which means that individuals have many employment opportunities (Andersson and Hellerstedt 2009). This could increase the likelihood of entrepreneurship because individuals know they have plenty of employment opportunities to fall back on if their own business would fail.

##### Heckman selection model - outcome equation

The dependent variable in the outcome equation is business income from the firm the entrepreneurs have started. In the baseline outcome equation, we include dummies for same industry spin-offs and occupational spin-offs. In addition to these two variables, we also control for age, sex, education, employer size, exit of establishment, MNE and a dummy for metropolitan regions. All these variables are expected to influence the success of the firm measured in terms of the business income it generates in its first year of operations.

We also include a number of controls that only pertain to those previous employees who chose to start a firm. These variables include dummies for whether the firm was started with any employees or as an incorporated firm. Both variables are expected to be positively related to the income derived from the firm. For example, starting incorporated new firms is costlier than starting self-proprietorships, and being incorporated may reflect more ambitious entrepreneurship (see e.g. Åstebro and Tåg 2015). Furthermore, familiarity with the local environment has been argued to influence positively the success of firms (Dahl and Sorenson 2012). Entrepreneurs build up local networks that they can exploit to the benefit of their firm. This potential effect is captured by a dummy variable, which indicates whether the entrepreneur starts the firm in the same local environment as the previous workplace. To check the robustness of the baseline model, we will also present two alternative specifications in which we include fewer controls in both the outcome and selection equation to avoid the possibility of ‘overcontrolling’ and ‘bad controls’ (cf. Angrist and Pischke 2008).

#### Survival in self-employment

To address success in terms of survival, we estimate a Cox hazard model for the longevity of the individual’s status as business owner. In this model, negative coefficient estimates indicate that the hazard of leaving the state of self-employment is lower. We include all variables in the outcome equation of the Heckman model.

## Results

The results from the Heckman selection model are presented in Table 4. We first discuss the baseline model (Model I) and then the results from Models II and III, which include fewer controls. In all models, the Mill’s lambda is significant, which indicates the importance of correcting for selection from employment into self-employment. Overall, results are in line with the expectations.

**Table 4:** Estimated effects on the probability of transcending to self-employment and on business income, Heckman selection model

	Model I		Model II		Model III	
	Outcome	Selection	Outcome	Selection	Outcome	Selection
Occupational spinoff	0.156***		0.164***		0.142***	
	(0.0255)		(0.0271)		(0.0276)	
Industry spinoff	0.0826***		0.238***		0.212***	
	(0.0287)		(0.0280)		(0.0304)	
Incorporated	0.687***					
	(0.0298)					
Employees	0.0850***					
	(0.0330)					
Stayer	0.00800					
	(0.0589)					
Age	0.0569***	0.0526***	0.128***	0.0634***	0.0563***	0.0613***
	(0.0113)	(0.00480)	(0.0123)	(0.00446)	(0.0125)	(0.00468)
Age_sq	−0.000641***	−0.000562***	−0.00139***	−0.000654***	−0.000614***	−0.000656***
	(0.000131)	(5.75e-05)	(0.000145)	(5.35e-05)	(0.000145)	(5.62e-05)
Male	0.178***	0.323***	0.480***	0.388***	0.145***	0.335***
	(0.0376)	(0.0162)	(0.0415)	(0.0146)	(0.0431)	(0.0152)
Highly educated	0.191***	0.0874***	0.463***	0.162***	0.227***	0.0501***
	(0.0332)	(0.0159)	(0.0345)	(0.0137)	(0.0322)	(0.0148)
Employer size 2004 (ln)	0.0139	−0.211***			0.0723***	−0.220***
	(0.0170)	(0.00463)			(0.0210)	(0.00458)
Establishment exit	0.0224	0.241***			−0.104**	0.234***
	(0.0453)	(0.0226)			(0.0491)	(0.0223)
MNE	0.130***	−0.141***			0.195***	−0.100***
	(0.0364)	(0.0150)			(0.0390)	(0.0146)
Metropolitan	0.123***	0.184***		0.0602		0.241***
	(0.0276)	(0.0421)		(0.0401)		(0.0413)
Tenure		−0.00517***		−0.0103***		−0.00650***
		(0.00136)		(0.00126)		(0.00134)
Management		0.411***				
		(0.0206)				
Specialist		0.356***				
		(0.0217)				
Qualified		0.160***				
		(0.0185)				
Office		−0.136***				
		(0.0301)				
Service		−0.0348				
		(0.0234)				
Wage income 2004 (ln)		−0.109***		−0.138***		−0.0299***
		(0.00974)		(0.00816)		(0.0102)
Mill’s lambda	−0.253***	**-**	0.906***	**-**	0.318***	**-**
# observations	3615	1,472,706	3615	1,472,706	3615	1,472,706

The table reports coefficients from a Heckman model estimated on a sample of 1,472,706 private sector employees in 2004. The selection equation estimates the influence the independent variables have on the probability that an individual switches from regular employment to full-time self-employment in 2005. There are 3615 individuals undertaking such a transition. The outcome equation estimates the influence on the log of business income of the individuals switching from employment to self-employment. The selection equation always includes dummies for industry belonging of the employer in 2004 (see Table 3)

* p < 0.10; ** p < 0.05; *** p < 0.01

For the selection equation regarding entering self-employment, we find a positive effect of age, although the square term suggests its impact is declining with age. Also, the education level has a positive effect, whereas firm tenure has a negative impact on the likelihood of starting a new firm. This is consistent with the interpretation that tenure proxies match quality and the build-up of firm-specific skills. Both raise the opportunity costs associated with leaving employment for entrepreneurship. We also show that employees with a university education and men are more likely to transition to self-employment.

Working in larger establishments and in establishments that are part of a multinational are negatively associated with the propensity of starting a business, which is in line with Hvide’s (2009) argument that bigger and international firms offer more opportunities for career development. It is also consistent with the jacks-of-all-trades argument that smaller firms prepare for entrepreneurship. The income earned in the previous year has a negative effect, indicating the opportunity costs associated with starting a firm. We also find that employees who in 2004 worked in an establishment that closed in 2005 are more likely to switch from employment to self-employment between 2004 and 2005, which suggests that they are ‘pushed’ into self-employment.

The results also confirm that there is significant heterogeneity between occupations regarding the probability of transitioning to entrepreneurship. Managers, specialist workers and qualified workers show higher propensities to leave employment for self-employment. The positive effect regarding managers is in line with the idea that they have skills associated with how to run a business, which may transfer to many different contexts. The increased likelihood of specialists and qualified workers starting a firm can be appreciated as reflecting the variety of contexts in which they can exploit their specialized skills. Their specializations in, for example, controlling, accounting, business law and IT defies the jack-of-all trades idea, but such ‘specialized skills’ are likely to be usable in a range of business contexts. All selection equations include controls for industry. These show, though not reported, that working in high-tech manufacturing and knowledge-based services is associated with higher propensity to switch to self-employment. This is consistent with the argument in the so-called ‘knowledge spillover theory of entrepreneurship’ (Acs et al. 2009), which claims that entrepreneurial opportunities are endogenous and are more frequent in knowledge-intensive contexts.

We now turn to the outcome model, which assesses the income generated from the business. Descriptive statistics show that the mean (median) log of business income is 7.85 (7.94) for occupational spinoffs and 7.60 (7.89) among other entrepreneurs. Is this difference driven by differences between characteristics and background of the two types of entrepreneurs or does it reflect an advantage for occupational spinoffs? The results in the table suggest that occupational spinoffs do indeed perform better than other types of entrepreneurs and that this is not simply an artefact of entrepreneurs having certain observable characteristics or coming from certain types of firms. Even after controlling for sample selection as well as characteristics of the individual, previous employer and other factors, we find that occupational spin-offs are positively associated with business success, as evidenced by a higher business income. We also confirm that industry-specific experience - industry spin-offs - positively influences the success in self-employment. In the baseline model, Model I, the effect is smaller than that of occupational spin-offs.

Two other results stand out. The size of the previous employer (MNE) have a positive effect on income, while the effect in the selection equation is negative. This suggests that opportunity costs of leaving large firms are relatively high. If employees are reluctant to leave large firms for self-employment, they are likely to only do this when a viable and profitable business opportunity arises. We also find that incorporated new firms, having employees and being highly educated is positively associated with business income. Furthermore, entrepreneurs operating in the metropolitan regions Stockholm, Göteborg and Malmö enjoy higher business incomes. Older self-employed tend to have higher business income, though the positive effect falls off as age increases. The positive effect of age on business income may be interpreted as an effect of general experience.

Models II and III exclude several control variables. Model I excludes employer characteristics and occupation dummies, as well as the indicators of whether the new firm has employees, is incorporated and whether the individual is a stayer or not. Model III adds employer characteristics in both the selection and outcome equation. The reason for estimating these reduced models is to ensure that our baseline results are not driven by the regression model being ‘overcontrolled’ and include so-called ‘bad controls’ (cf. Angrist and Pischke 2008). It is evident from these models that the main results are robust and hold. However, in Models II and III, the influence of occupational spinoffs is in quantitative terms smaller than the influence of industry spinoffs. Still, the effect of being an occupation spinoff is positive and statistically significant.^7^

**Fig. 1:**
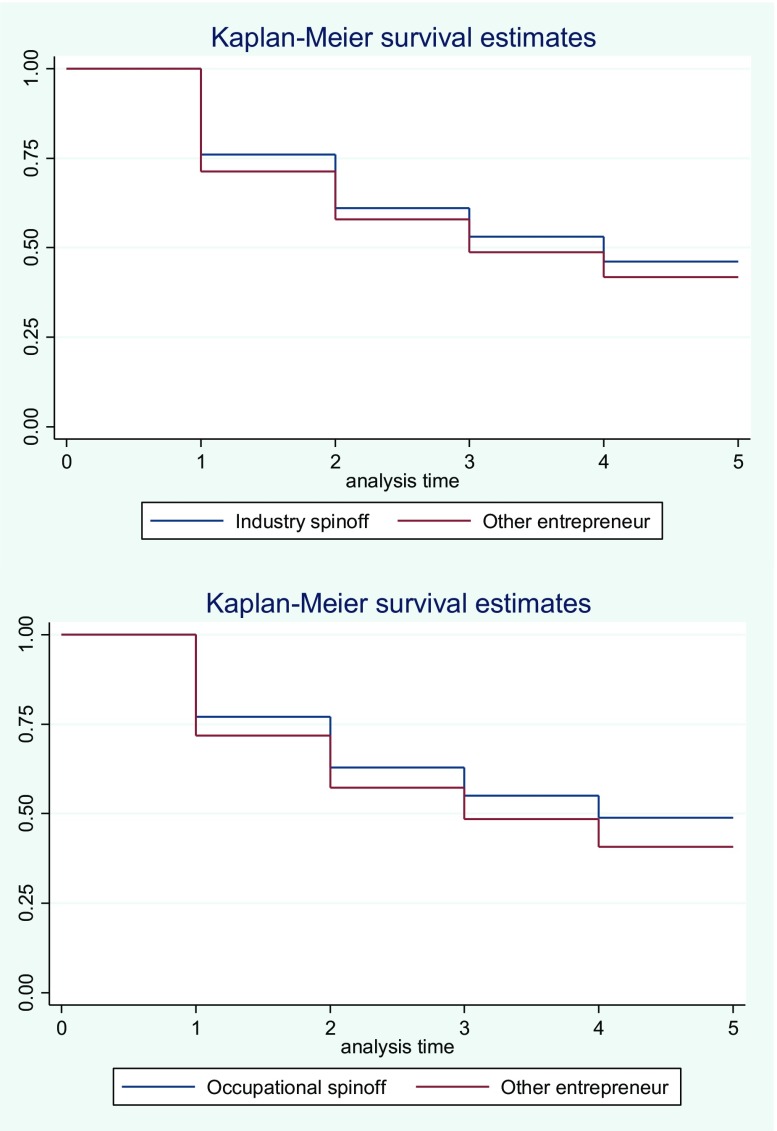
Survival as self-employed. Upper panel: industry spinoffs compared to other self-employed. Lower panel: occupational spinoffs compared to other entrepreneurs

To further substantiate the results, we repeat the analysis using a different indicator of entrepreneurial success, i.e. the longevity of the entrepreneurship spell. Figure 1 presents Kaplan-Meier survival estimates for the longevity of the self-employed as business owners. The left panel compares industry spinoffs to other entrepreneurs (i.e. same industry) and the right panel compares occupational spinoffs to other entrepreneurs. It is clear from the figure that both industry and occupational spinoffs show higher survival rates as compared to other entrepreneurs, though occupational spinoffs appear to have a somewhat higher survival rate than industry spinoffs.

Table 5 presents estimates of a Cox hazard model allowing us to test the influence that our variables of main interest have on individuals’ longevity as entrepreneurs. A negative sign of a coefficient estimate indicates that the variable in question has a negative influence on the hazard of leaving the state of self-employment. The explanatory variables are the same as in the outcome equation in Table 4. Model A includes only the industry and occupation spinoff variables. Models I, II and III are the same as the outcome equation in the corresponding models in Table 4.

**Table 5:** Estimated effects on the longevity as entrepreneur, Cox hazard model 2005–2009

	Model A	Model I	Model II	Model III
Industry spin-off	−0.0745	−0.0455	−0.0583	−0.0726
	(0.0464)	(0.0521)	(0.0467)	(0.0509)
Occupational spin-off	−0.182***	−0.221***	−0.214***	−0.214***
	(0.0462)	(0.0474)	(0.0472)	(0.0473)
Highly educated		0.195***	0.216***	0.223***
		(0.0516)	(0.0497)	(0.0503)
Incorporated		−0.118**		
		(0.0537)		
Employees		−0.119*		
		(0.0615)		
Stayer		−0.216**		
		(0.0989)		
Age		−0.0280	−0.0336*	−0.0328*
		(0.0189)	(0.0187)	(0.0188)
Age_sq		0.000282	0.000342	0.000333
		(0.000224)	(0.000222)	(0.000222)
Male		−0.143**	−0.164***	−0.163***
		(0.0572)	(0.0568)	(0.0569)
Employer size 2004 (ln)		0.00170		−0.00397
		(0.0156)		(0.0154)
Establishment exit		0.0987		0.0907
		(0.0755)		(0.0735)
MNE		−0.0443		−0.0348
		(0.0653)		(0.0649)
Metropolitan		0.143***		
		(0.0460)		
# observations	3615			

The table reports coefficient from a Cox proportional hazard model. The reported estimates are show the influence the independent variables have on the hazard in terms of longevity as entrepreneurs among individuals who switched from full-time employment to full-time self-employment in 2005. There are 3615 individuals undertaking such a transition

* p < 0.10; ** p < 0.05; *** p < 0.01

The coefficient for occupational spin-offs in explaining the hazard of exit out of self-employment is negative, which translates to a greater longevity of occupational spin-offs compared to other self-employed. This is consistent with the idea that a sharp correspondence between occupational background and industry of choice of an entrepreneur breeds success. No effect is found for industry spin-offs in this analysis. This result holds up across the different specifications. The estimated impact of the control variables is in line with expectations.

Older entrepreneurs with presumably more general experience appear to have greater longevity, though the effect is not statistically significant in Model I. Higher educated entrepreneurs show lower longevity, which can be interpreted as a reflection of the opportunities on the labor market available to them. They are thus more likely to leave entrepreneurship for employment. The positive association between being in a metropolitan region and hazard (Model I) could be given a similar interpretation, although it can also reflect more intense competition between businesses. Entrepreneurs who stay in their home region to start a business (stayer) also show longer longevity as entrepreneurs (Model I). This is consistent with the idea that entrepreneurs operating in their home region could use local ties and networks in their business operations (Dahl and Sorenson 2012). A further finding is that men show greater longevity as entrepreneurs than women. We find no statistically significant influence the size of the previous employer, whether the previous employer is a multinational firm or whether the establishment at which the individual previously worked exited or not.

## Conclusions

Labor market experience is a crucial aspect of entrepreneurial success. Relevant experiences, materialized in, for example, professional networks and skills, can be used to the benefit of the firm. Departing from this basic idea, an important issue is then to identify relevant contexts for the development of experiences that can be exploited in entrepreneurship. Existing studies have foregrounded experience in the same industry as an important context for learning. Spin-off studies stress the correspondence between the industry entrepreneurs in which previously worked and the industry in which they started their firm. The typical finding is that a correspondence between industries has a positive effect on entrepreneurial success. This study argues that, in addition to industries, the development of relevant skills and experiences can also be specific to occupations. We empirically show that a correspondence between specific occupations and specific industries is a relevant factor in explaining entrepreneurial success.

The effect of occupational background is found to be twofold in our study of Swedish entrepreneurs who transition from having only a job in wage employment to being only self-employed. First, certain occupations prepare people for entrepreneurship per se, because the occupations involve a skillset that can be used in the set-up and management of a new firm. In the sample of Swedish entrepreneurs studied here, managers, specialists and qualified workers are found to be likely to start businesses, compared to other occupations. Second, we find evidence that certain occupation-industry transitions are beneficial for entrepreneurial success. This suggests that occupations provide a learning context for skills that can be exploited most beneficially in certain industries. In the analysis, such combinations are defined by identifying the most common start-up industry given a certain occupation. Start-ups in the most frequent occupation-industry combinations are labeled occupational spin-offs. This study empirically shows that, over and above general occupation and industry effects, entrepreneurs starting occupational spin-offs enjoy relatively high incomes from self-employment. In addition, the hazard of exiting self-employment is lower for entrepreneurs in an occupational spin-off than for other entrepreneurs. These effects hold after controlling for a full set of additional factors that explain entrepreneurship propensity and success.

We interpret the findings as a sign that, albeit relevant, industry-experience masks a considerable heterogeneity in tasks and specific skills that are at least partially captured in classifications of the occupation. As such, in the study of entrepreneurial outcomes and intentions, we maintain that not only industry-industry transitions are important to account for relevant learning. Observed occupation-industry transitions also contain meaningful information on contexts of entrepreneurial learning and the successful exploitation of the skills and experiences developed. With this insight, the study contributes to the literature on labor market experiences and entrepreneurship intensions and outcomes. In a more general sense, however, the study also speaks to the wider literature on skills in regional labor markets and their impact on regional economic restructuring and development. The study reinforces recent arguments that stress the importance of the nature of local labor markets and skill composition of the labor market force available. Although part of the regional skill composition is represented in the industry structure, the occupational structure is of significant influence as well.

## Footnotes

1 In discussing the general assumptions behind his model of entrepreneurship, Lucas (1978 p.510) writes: “As a preliminary matter, it is necessary to decide whether to follow Viner and develop a model at the *industry* level, or to theorize at an economy-wide level. The former would be appropriate if managerial ability were industry-specific, as a result of “nature” or of the accumulation of industry-specific expertise. There is no doubt that these considerations matter, at some level, but the multi-product of at least the largest modern firms and the mobility of top managers across industries suggest that, of one is choosing between these extremes, it is best to attack the problem at an economy-wide level.”

2 Even though the sample selection is aimed at reducing other sources of experiences, the sampling frame is left-censored, which prevents us from observing heterogeneity in prior labor market experiences. This could influence the magnitudes of the effects of immediate experiences. However, there is no reason to suspect that the unobserved prior experiences would impact differently on industry spin-offs or occupational spin-offs, the relative impact of both types of experience being the important argument in the study.

3 The data do not allow relating occupations in employment to occupations in self-employment directly because self-employment is itself an occupation in the data. It is not further differentiated so we cannot distinguish distinct occupations within the group of self-employed on which to base the link. Instead, we use occupations as employee and industries as self-employed for the measure of revealed relatedness.

4 In the context of inter-industry labor flows, Neffke and Henning (2013) use a relative measure of relatedness, corrected for industry size, growth and wage level. This is appropriate because employment transitions are contingent on the availability of jobs and their characteristics, i.e. the jobs are exogenously determined for employees. Flows to an industry are thus likely to be dependent on the opportunities to find a job in that industry. In the context of entrepreneurs, the choice of industry is not constrained in the same way as it is for employees, since they employ themselves through the creation of a new firm. Job creation is thus endogenous in the process and not directly dependent on, for example, the size of the industry. Therefore, we argue that relatedness between occupations and industries can effectively be established using the magnitudes of the flows.

5 Technically, the second stage equation includes Mill’s lambda defined as *λ* = *ϕ*((**x** *β*)/Φ(**x** *β*)) where *ϕ*(.)is the density function of the standard normal distribution and Φ(.) is the cumulative normal distribution coming from the first stage model.

6 To account for the skewed distribution of Age, but also Age squared, years of tenure, wage and size of the employer, these variables enter the models in logged form.

7 We have also undertaken two additional robustness tests. First, we estimated the models by excluding the same-industry dummy in the outcome equation and it does not alter the significance of the occupational dummy. Second, we ran the models when excluding individuals who, as employed, had occupations that are more tightly connected to specific industries, such as “service workers and shop sales workers” and “skilled agricultural and fishery workers”. This is to make sure that our results regarding occupational spinoffs are not driven by the set of occupations with strong links to specific industries. In both instances, our results remain robust. These additional estimations are available from the authors upon request.
